# Dose Effect Evaluation and Therapeutic Window of the Neuro-EPO Nasal Application for the Treatment of the Focal Ischemia Model in the Mongolian Gerbil

**DOI:** 10.1100/2012/607498

**Published:** 2012-05-22

**Authors:** Iliana Sosa Teste, Yuneidys Mengana Tamos, Yamila Rodríguez Cruz, Adriana Muñoz Cernada, Janette Cruz Rodríguez, Nelvis Subirós Martínez, Rosa Maria Coro Antich, Alina González-Quevedo, Julio Cesar García Rodríguez

**Affiliations:** ^1^National Center for Laboratory Animal Breeding, Havana, Cuba; ^2^Histology Department, Preclinical and Basic Science Institute, Havana, Cuba; ^3^Center of Molecular Immunology (CIM), Havana, Cuba; ^4^Center of Research and Development of Medicaments (CIDEM), Havana, Cuba; ^5^Institute of Neurology and Neurosurgery (INN), Havana, Cuba

## Abstract

Cerebrovascular disease is the third leading cause of death and the leading cause of disability in Cuba and in several developed countries. A possible neuroprotective agent is the rHu-EPO, whose effects have been demonstrated in models of brain ischemia. The Neuro-EPO is a derivative of the rHu-EPO that avoids the stimulation of erythropoiesis. The aim of this study was to determine the Neuro-EPO delivery into the central nervous system (CNS) to exert a neuroprotective effect in cerebral ischemia model of the Mongolian gerbil. The Neuro-EPO in a rate of 249.4 UI every 8 hours for 4 days showed 25% higher viability efficacy (*P* > 0.01), improving neurological score and behavior of the spontaneous exploratory activity, the preservation of CA3 areas of the hippocampus, the cortex, and thalamic nuclei in the focal ischemia model of the Mongolian gerbil. In summary, this study, the average dose-used Neuro-EPO (249.4 UI/10 **μ**L/every 8 hours for 4 days), proved to be valid indicators of viability, neurological status, and spontaneous exploratory activity, being significantly lower than that reported for the systemically use of the rHu-EPO as a neuroprotectant. Indeed, up to 12 h after brain ischemia is very positive Neuro-EPO administration by the nasal route as a candidate for neuroprotection.

## 1. Introduction

Till now, there is not a drug sufficiently effective, specific, and of safe access to the central nervous system (CNS) that could be used as a neuroprotectant in stroke in acute or chronic stage. Furthermore, most effective neuroprotective therapeutic agents with positive preclinical results in brain ischemia animals models are not tolerated clinically [1, 2]. To use the same molecules that are expressed in the brain after the stroke [3] helping to the endogenous brain protection, may be our strategy for finding a solution to this difficulty. One of these molecules is the recombinant human erythropoietin (EPO). Recombinant variant (rHu-EPO) has been evaluated for these purposes in different models of stroke such as in mouse, rat, gerbil, and rabbit, including global and focal cerebral ischemia [4–9], where it has shown convincing neuroprotective effect.

Although the neuroprotective mechanism of rHu-EPO is still investigated, it has been argued that its action is mediated by receptors found on the walls of vascular endothelium and in astrocytes [10, 11]. The mechanisms of rHu-EPO as a neuroprotective agent appear to be multifactor. The rHu-EPO can indirectly mediate as neuroprotectant on the restoration of blood supply to tissue damage or acts directly on neurons by activation of several molecular signaling pathways [12].

The neuroprotective effects of rHu-EPO may be produced by different factors within that included: the antagonism of glutamate-induced cytotoxicity, the increased expression of antioxidant enzymes, the decrease of the formation of nitric oxide (NO)-mediated free radicals [13], the normalization of cerebral blood flow [14], the influence neurotransmitter release, the promotion of angiogenesis [15, 16], the inhibition of apoptosis induced by excitotoxins and nitric oxide, the increased expression of antiapoptotic genes, the neurotrophic effect, the decrease of the neuronal excitability, the cerebral anti-inflammatory effect, the inhibition of kainate-induced apoptosis and the pro-inflammatory cytokine production, and the neurogenic and angiogenic effect that contributes to ischemic preconditioning; all together can justify the use of rHu-EPO in the treatment of cerebrovascular, neurodegenerative, and psychiatric diseases [15, 17].

The organs with narrow endothelial barriers, as the CNS, generally exclude polar and high-weight molecules [18, 19]. However, for certain proteins, a transit mechanism mediated by receptors, such as transferrin, insulin, and leptin has been established [20]. This same mechanism has been proposed to explain the brain delivery of the rHu-EPO applied intravenously. This hypothesis is based primarily on the fact that rHu-EPOA administered systemically can be detected in the CSF of rats, sheep, and monkeys in neuroprotective concentrations, with a delay of, approximately, 60 minutes [21–24]. Second, immunohistochemical studies have demonstrated a high density of EPO-R around the brain capillaries, manly in astrocytes feet and on the luminal side of the capillary endothelial cells [6, 25].

Repeated administration of rHu-EPO high doses can activate the hematopoietic process with the potential risk of increasing blood viscosity, which might worsen the outcome after stroke either because of hematocrit elevation or other negative effects [26]. Indeed, transgenic mice, with systemic over expression of rHu-EPO, were observed; they presented hematocrit levels above 80% and developed longer strokes, compared to their nontransgenic siblings, despite the high expression levels of EPO in the brain [27].

In a previous report, we have demonstrated that intranasal (*IN*) administration of Neuro-EPO proved to be safe, reaches the brain in few minutes, does not stimulate erythropoiesis after acute treatments, and shows efficacy in some rodent models of brain ischemia [28].

Neuroprotection is based in the pharmacological prevention or limitation of an ischemic cascade progression form in the brain tissue once it has begun [2]. The therapeutic window is the time during which the drug may show effectiveness of and depends on the time course of biochemical changes affecting the treatment [29]. The treatment dosing regimen and duration must be tailored to the biochemical process to be affected and must also take into account differences in drug metabolism between species [30].

The objective of this work was both to determine the dose response and the therapeutic window with the Neuro-EPO *IN* application in the Mongolian gerbil focal ischemia model, using evaluating variables such as viability, neurologic deficit score, spontaneous exploratory activity, and histopathological status.

## 2. Materials and Methods

### 2.1. Animals

 One hundred and forty male Mongolian gerbils (12–15 weeks; 70–90 g) were provided by the National Center for Laboratory Animal Breeding (CENPALAB) in Havana, Cuba and were adapted to experimental conditions for 7 days. The animals were maintained in controlled environmental rooms at 22 ± 2°C, relative humidity 55–60%, light-dark cycle was 12 h/12 h, and there were 15–20 room air changes per hour.

All materials used to maintain the animals were autoclaved at 121°C for 20 min. Food and water was provided *ad libitum*. Each protocol was discussed and approved by the Institutional Ethics Committee, considering that the international standards established by CCAC [31] *rHu-EPO* (ior CIM from CIMAB SA, and CIDEM Havana, Cuba) and Neuro-EPO (not a commercial product; patent PCT/cu2006/000001 Patent 20050138) were supplied by the Center of Molecular Immunology (CIM) Havana, Cuba and diluted in PBS (pH 7.0) at 0.15 mM. Intranasal administration was performed essentially as described previously [32] taking into account the established *Guide to the Care and Use of Experimental Animals. *Immediately after surgery, the gerbils were placed on their backs, and a total of 10 *μ*L of Neuro-EPO or a corresponding volume of vehicle solution per gerbil was given in nose drops of (5 *μ*L/drop) over a 1- to 2-min period, alternating drops between the left and right nares. The mouth and the opposite naris were closed during the administration, so the drops could be naturally inhaled high into the nasal cavity.

### 2.2. Surgical Procedure and Spontaneous Exploratory Activity Measurement

Gerbils were anesthetized with ketamine atropine-diazepam (47; 0.02; and 5 mg/kg, resp.). Lesions were performed according to Butterfield and McGraw's method [33]. Briefly, the right common carotid artery (CCA) was isolated and double-ligated using silk 5-0 suture and was sectioned with surgical microscissors. In the controlled operated animals, the artery was only isolated.

Twenty-four hours after unilateral permanent ischemia, the appearance of the following clinical signs of infarction was assessed: palpebral ptosis, bristling, loss of tone and reflexes in the four limbs, postural asymmetry, rolling or circling, and death. Each sign was scored separately. The sum of scores creates a general neurological score for each animal. The clinical signs of brain infarction were assessed as previously established [34]. In brief, each animal was evaluated to determine its neurological state according to a scale (with a maximum of 30 and a minimum of 0) [34].

Gerbils were placed in the center of a round open field (30 cm diameter and 25 cm height). “Rearing” was considered as standing straight up on the hind limbs and tail, until the animal returned any forelimb to the floor or touched the open field wall with any forelimb. Neither a subsequent straight after a rearing without reaching the floor with any forepaw nor kangaroo-like posture was considered. Exploratory activity was determined by the rearing counted at 3, 6, and 9 min in the open field. An average of the total rearing counts was calculated for each group, rendering three dots per trial. Dots were plotted, and the line obtained by the minimal square method was calculated and considered as the habituation curve. The slope of the habituation curve was used to characterize the animal's state.

### 2.3. Experimental Animals Groups

The distribution of the experimental groups to study dose. (In each group *n* was 35 animals.)

(i) Control up (false injured).
(ii) Injured group with application of the vehicle (placebo).
(iii) Ischemic Group treated with Neuro-EPO in a low dose, 0.5 mg/mL of Neuro-EPO concentration (24.9 UI every 8 hours for 4 days).
(iv) Ischemic Group treated with Neuro-EPO in a medium dose, 1 mg/mL of Neuro-EPO concentration (249.4 UI every 8 hours for 4 days).
(v) Ischemic Group treated with Neuro-EPO in a high dose, 2 mg/mL of Neuro-EPO concentration (2 499.4 UI every 8 hours for 4 days).

We assessed survival, neurological status, and spontaneous exploratory activity at 0 and 7 days. The histological study, to evaluate the effect of the drug on the tissue, was performed at the dose where the best behavioral performance was obtained.

The distribution of the experimental groups to study the therapeutic window (treated with Neuro-EPO in a medium dose of 1 mg/mL concentration (249.4 UI every 8 hours for 4 days)) in each group *n* was 35 animals.

(i) Control group (false injured).
(ii) Ischemic Group with application of vehicle *IN* (placebo).
(iii) Ischemic Group with Neuro-EPO application *IN* immediately after injury.
(iv) Ischemic Group with Neuro-EPO application *IN* starting at 6 hours after injury.
(v) Ischemic Group with Neuro-EPO application *IN* starting 12 hours after injury.
(vi) Ischemic Group with Neuro-EPO application *IN* starting 18 hours after injury.

### 2.4. Tissue Preparation

#### 2.4.1. Histology

 Gerbils were anesthetized for cardiac perfusion. Each animal was perfused with 20 mL of saline solution and fixed with 60 mL of 4% buffered formaldehyde solution, pH 7.0. Brains were carefully removed and postfixed in the same solution for several days. Fixed brains were dehydrated and embedded in paraffin. Serial coronal sections of 8-*μ*m width were obtained. Sections were stained with hematoxylin eosin and described using light microscopy.

#### 2.4.2. Quantitative Morphological Analysis

Preparations containing dorsal hippocampus (from 1.3 to 1.6 after Bregma), according to the atlas by Loskota et al. [30], were used. Observers were blind to the preparation origin. Pictures of 400 times magnifying power were digitized and processed by the Image J program (http://rsb.info.nih.gov/ij/, NIH). The area of hippocampus CA1 pyramidal layer was measured, subtracting the area occupied by pyknotic cells. Then, the normal remaining area of CA1 subfields was determined.

#### 2.4.3. Statistical Analysis

 The log rank test using GraphPad Prism 4 to evaluate survival percentage for the three treated groups and control was employed. Frequencies of clinical signs and rearing counts were analyzed employing the Chi-square independence test. The Mann-Whitney *U* test was used for the comparison between the control and other groups of neurological score rearing counts, slope, and remaining CA1 area. The Wilcoxon matched pair test was used for comparisons between hemispheres. For correlations between morphological and functional variables, the Spearman's rank correlation coefficient was used. The statistical analysis was carried out according to the Microsoft STATISTICA version 6.0 program. In all, a level of significance of *P* < 0.05 was accepted.

## 3. Results

**Figure 1:**
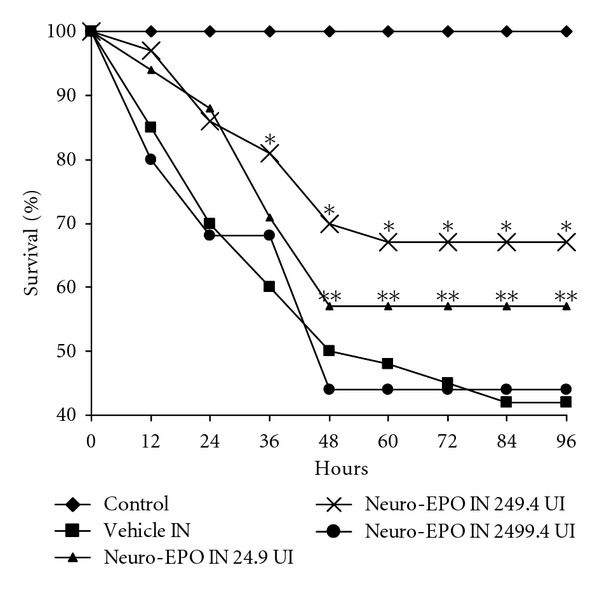
Percent of survival in ischemia model in Mongolian gerbil, evaluating different doses of intranasal formulation of Neuro-EPO *(*P* < 0.01); **(*P*,0.02) indicate significant difference in Kaplan Miller Test respect with vehicle (*n* = 35 per group).

Previous studies have shown that *IN* Neuro-EPO had neuroprotective effect in a similar ischemia animal model [35]. Percent of viability was evaluated at three different doses of *IN* Neuro-EPO. The best results were detected at 24.9 and 249.4 UI both significantly higher when compared with the control group. The dose that showed the best percent of viability (67%) when was compared with the injured group treated with *IN* vehicle was the 249.4 UI (Figure 1).

**Figure 2:**
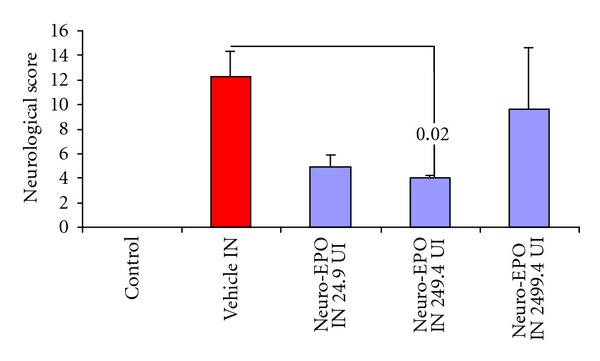
Clinical signs of stroke at 24 h of permanent occlusion of the right common carotid in the Mongolian gerbil, evaluating different doses of the intranasal formulation of Neuro-EPO. Numbers indicate significant difference between bars in the *U* test of Mann and Whitney in respect with vehicle. (*n* = 35 per group).

The only dose for *IN* Neuro-EPO that showed a significant effect on the neurological score (Figure 2) with respect to the vehicle was the median dose of (249.4 UI).

**Figure 3:**
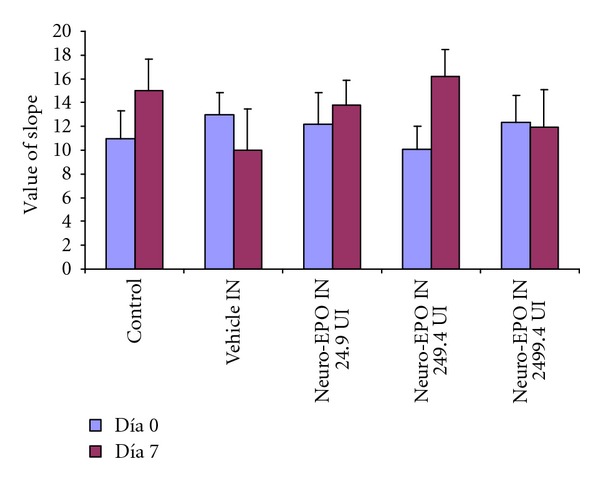
Values of slopes of the curve of room in the open field test, the day before and 7 days after surgery using different dosages of Neuro-EPO IN. Bars represented means ± standard deviation. *P* values in the Wilcoxon test. The comparison was carried out in each group among the day 0 and 7 days later. (*n* = 35 per group).

In the open-field behavioral test to measure learning at 7 days (Figure 3) ischemic animals treated with Neuro-EPO in the medium (249.4 UI) and low (24.9 UI) doses, a pattern of habituation similar to controls was shown. The animals in the vehicle-treated group showed poorer learning. This loss of habituation is interpreted as the inability to recognize the place already explored before carotid occlusion.

**Figure 4:**
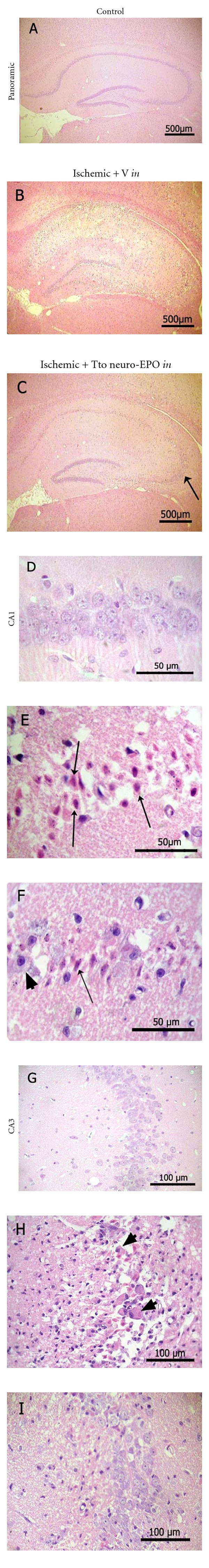
Model of focal ischemia. Dorsal hippocampus. Causes brain ischemia in the hippocampus at 7 days after occlusion (B, E, H). The Neuro-EPO protects the dorsal hippocampal CA3 sector in this model (C, I) and does not protect CA1 (F). H-E. A, B, C: Panoramic view. Arrow: conservation of CA3 neurons. D, E, F: Dorsal hippocampal CA1 sector. Arrows withdrawn neurons with pyknotic nuclei and eosinophilic cytoplasm. G, H, I Sector CA3 of the hippocampus. Arrowheads in E, F, and H macrophages with vacuolated cytoplasm.

**Figure 5:**
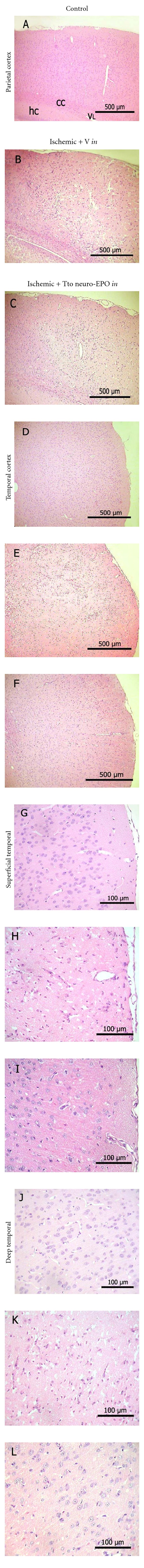
Model of focal ischemia. Parietal and temporal cortex. Unilateral ischemia produces infarction in the parietal cortices (B) and temporal (E, H, K). The Neuro-EPO rescues neurons in the temporal cortex (F, I, L). H-E. A, B, C: Panoramic view of the parietal cortex. hc: hippocampus; cc: corpus callosum; VL: lateral ventricle. D, E, F: A panoramic view of the temporal cortex. G, H, I: outer layers of the temporal cortex. H: Loss of the neural elements with holes. I: looks like the control. J, K, L: deeper layers of the temporal cortex. K: Loss of the neural elements with holes. L: looks like the control.

**Figure 6:**
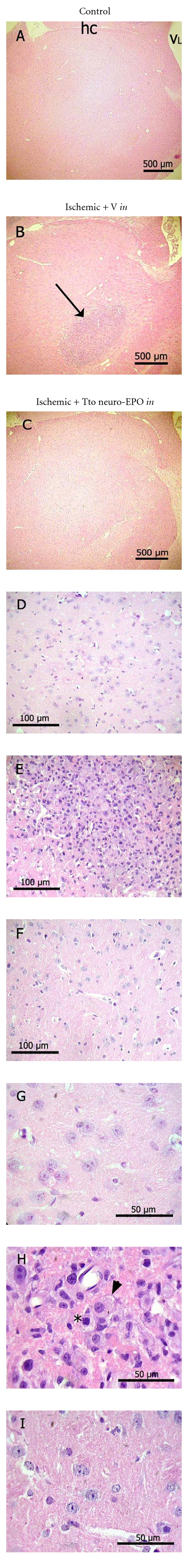
Model of focal ischemia thalamus. Unilateral ischemia produces large clusters of macrophages in the nuclei of the thalamus (B, E, H). The thalamus damage decreases with Neuro-EPO in (C, F, I). H-E. A, B, C: Panoramic view. Arrow: focus of macrophages. VL: lateral ventricle; hc: hippocampus. D, E, F: ×400 thalamus. E: Focus of macrophages. F: Control-like appearance. G, H, I: Thalamus x1000. H: Macrophage with vacuolated cytoplasm (arrowhead), macrophage in mitosis (asterisk). I: Looks like the control.

Ischemic animals treated with Neuro-EPO in the medium dose (249.4 UI) showed no neuroprotective effects in the CA1 region of the hippocampus; this is corroborated with the study of neuronal densities. This area of the hippocampus is one of the most sensitive to ischemia, and the neuroprotective effect of any drug would have less chance to be evidenced than less vulnerable areas. The histological involvement was minor in the areas of CA3, cortex, and thalamic nuclei in animals treated with Neuro-EPO as illustrated in Figures 4, 5, and 6.

Arteries are not frequently affected in ischemic injury, are on the periphery of the infarct, and supply these structures where neuroprotection was observed. In this group, there was only light damage in the CA1 and CA2 regions of the hippocampus.

Seven days after ischemia, the histological damage decreased in the above areas. The animals in the vehicle-treated group showed necrosis of the temporal and parietal cortices, hippocampus, thalamus, and caudate putamen, loss of CA1 pyramidal neurons and cognitive impairment. Infarction areas in the hemisphere ipsilateral to carotid occlusion in the animals treated with the vehicle were observed. On the contrary, these negative neurologic effects were reduced in animals treated with *IN* Neuro-EPO. The results of this work constitute an indirect evidence of the passage of Neuro-EPO to the CNS through the nasal cavity.

**Figure 7:**
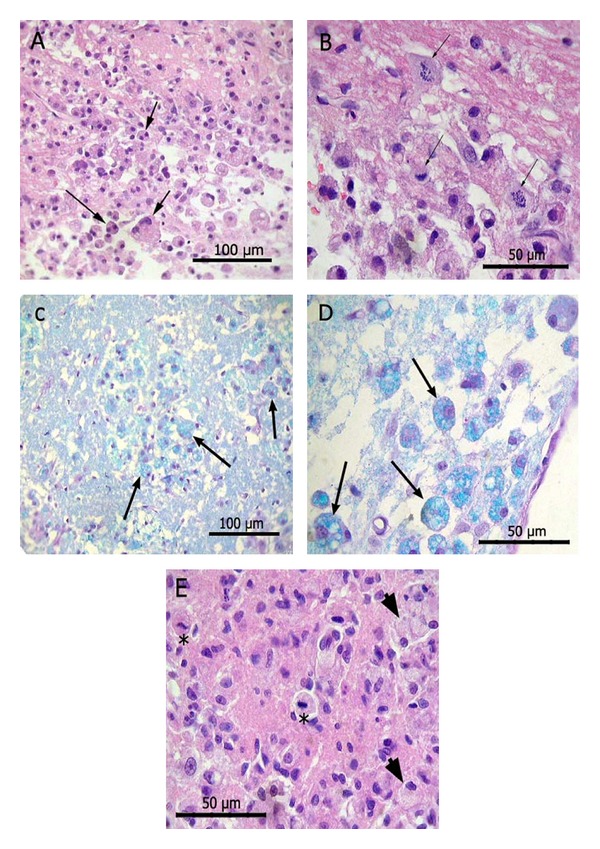
Macrophages in ischemic animals. A: Binucleate macrophages in the corpus callosum. H-E. B: Macrophages in mitosis in the corpus callosum. H-E. C: Macrophages in caudate putamen. Luxol fast blue y PAS. D: Macrophages near the lateral ventricle. Luxol fast blue y PAS. E: Macrophages with vacuolated cytoplasm in mitosis (asterisk) in thalamus. H-E. Model of focal ischemia. Macrophages (arrows) in several brain regions of ischemic animals.

Hippocampal CA1 pyramidal neurons showed that pyknotic, retracted, and acidophilic cytoplasm was observed (Figure 4). In the infarcted area, granule-fatty corpuscles (macrophages) filled with breakdown products of myelin, were observed.  Figure 7 clearly illustrates macrophages, some binucleate and mitotic processes. All these findings are characteristic, and they have been described by other authors in the ischemia model in injured animals [15, 36, 37]. 

**Figure 8:**
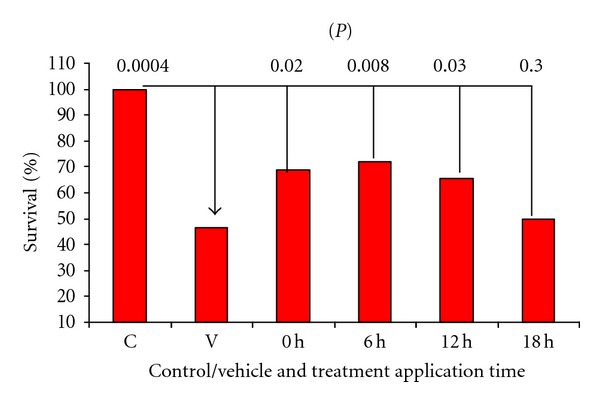
After 7 days of models of focal ischemia. *P* = Significance with regard to vehicle, indicating a significant difference in the *U* test of Mann and Whitney. (*n* = 35 per group). Dose Neuro-EPO *IN* 249.4 UI.

To determine the therapeutic window, groups of animals began receiving treatment with* IN *Neuro-EPO immediately after surgery at 0, 6, and 12 hours. These three groups showed a greater survival at 7 days after occlusion, when compared with the groups that received vehicle (Figure 8).

**Figure 9:**
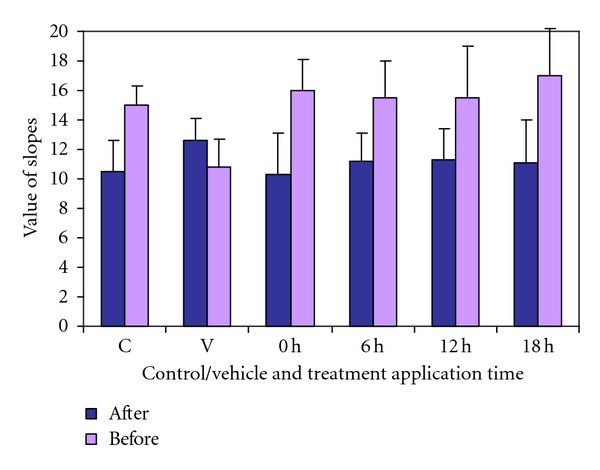
Values slope the day before and 7 days after models of focal ischemia. *P* values in paired rank test of Wilcoxon. (*n* = 35 per group) Dose Neuro-EPO *IN* 249.4 UI.

The comparison between the values of outstanding open-field test in the control group not subjected to ischemia showed significantly more negative values in the second determination, indicating a faster decline of the steep at that time (Figure 9). Similar result was observed in the groups treated with Neuro-EPO for 4 days, whether they were treated immediately or at 6, 12, or 18 hours after occlusion. However, vehicle-treated animals showed similar slopes within 7 days of ischemia.

**Figure 10:**
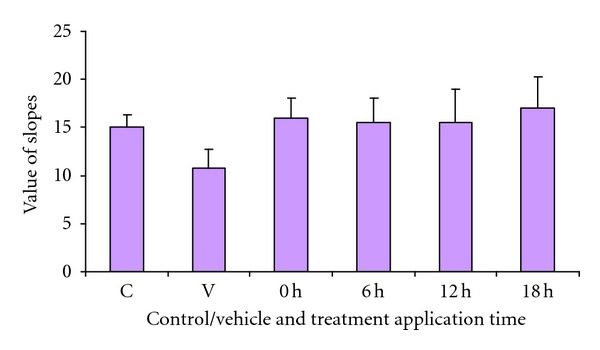
Slope values 7 days after of models of focal ischemia. *P* values of comparison against the vehicle group by the *U* test of Mann and Whitney. (*n* = 35 per group). Dose Neuro-EPO *IN* 249.4 UI.

Initiating treatment with the *IN* Neuro-EPO at 0, 6, 12, and 18 hours after the ischemic lesion showed effectiveness on the open field test, when compared with the vehicle (Figure 10).

**Figure 11:**
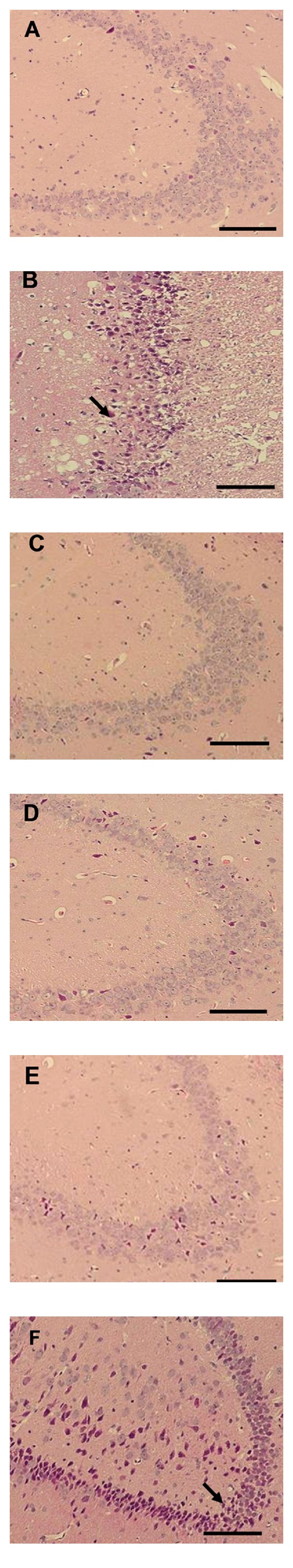
Hematoxylin-eosin. The Neuro-EPO protects the dorsal hippocampal CA3 sector in the focal ischemia model, (C, D, E). Bar 100 *μ*m A: dorsal hippocampal CA3 sector in control. B: Arrows withdrawn neurons with pyknotic nuclei and eosinophilic cytoplasm animals treated with vehicle. C, D, E: dorsal hippocampal CA3 without injury starting treatment with Neuro-EPO intranasal at immediately, 6 hours at 12 hours, respectively. F: Dorsal hippocampal CA3 sector starting treatment at 18 hours. Arrows withdrawn neurons with pyknotic nuclei and eosinophilic cytoplasm.

Neuro-EPO protects the dorsal hippocampus CA3 sector in the focal ischemia model (Figure 11). In the ischemic group animals treated with vehicle, an infarct area was observed in the hemisphere ipsilateral to carotid occlusion. Stroke area was identified by the death of most of the cellular elements, demyelination, presence of cellular debris, and partially empty areas. The lesion zone extended to all regions of the hippocampus (including much of the dentate gyrus) the parietal and temporal cortex, in the thalamic nuclei and caudate putamen. At 6 hours, the treated animals showed no histological damage in either group. In the CA3 area hippocampal pyramidal neurons showed pyknotic, retracted, and acidophilic cytoplasm at 18 hours, evidencing improvement in those treated with EPO.

## 4. Discussion

The focal ischemia model by carotid artery occlusion in Mongolian gerbils has been used to study the pathophysiology of focal cerebral ischemia and the evaluation of potential neuroprotective agents [38–40]. The mortality in this model reflects the severity of the injury, and it is an important variable in assessing drugs for the treatment of ischemia. In this model, mortality rates between 30 and 40% at 24 and 48 hours after ischemia have been reported [41, 42]. This allows us to suggest that these animals have a cognitive dysfunction induced by ischemia, which affected their learning and short-term memory [43]. In ischemic animals histological lesions found are similar to those described in other studies [44, 45]. Whereas ischemic animals showed no histological injury in neurological impairment and cognitive ability; one might think that the brain tissue, albeit with HE staining was observed intact, was biochemically committed [46].

It has been reported that in the penumbra area there is still a cerebral brain fluid (CBF) below normal levels [47]. Perhaps the arrival of Neuro-EPO to this penumbra region protected these cells against excitotoxicity and phenomena such as apoptosis. It has also been shown that rHu-EPO and its derivatives stimulate the process of angiogenesis and neurogenesis, providing a favorable microenvironment for neural plasticity during stroke recovery [48–50].

Comparison of the treated and untreated injured groups was carried out to assess the learning capacity linked to short-term memory, dependent on synaptic transmission and neuromodulation. According to our results, treatment with *Neuro-EPO IN* equally protects against the occurrence of dysfunction in learning and short-term memory, even starting the treatment at 18 hours, suggesting a neuroprotective effect on this functional impairment.

The effects observed in the preservation of habituation behavior in animals, where treatment started as late as 18 hours, suggests the inhibition of processes such as neuronal apoptosis in brain regions associated with that behavior, and the expression of neurotrophic properties that enhance neuronal function. Apoptosis requires the expression of effector proteins in the longer term; therefore, it could be interfered with at 18 hours after onset of ischemia by Neuro-molecules such as EPO, which can induce the expression of antiapoptotic proteins [48]. All these mechanisms strongly point in favor of the process of neuroplasticity that may be induced by Neuro-EPO in the damaged brain.

The Hippocampal CA1 region shows severe neuronal loss in 85–95% of the gerbils after 7 days of occlusion. It has been shown that neuronal death in the case of ischemia is selective for neuronal groups; besides, neuronal death does not occur either in the same manner or at the same time. There are cell groups such as the vulnerable Hippocampal CA1 pyramidal cells that begin to die from the first 5 minutes after ischemia and others such as the CA2 and CA3 cells, which the process of neuronal death can take about 6 to 12 hours to begin [51].

The results suggest that despite the fact that permanent occlusion did not induce infarction in all animals, as described in the literature, there is a decrease in cerebral blood flow that could induce damage, which becomes evident in the chronic phase, when secondary degeneration processes have already taken place [52, 53]. It has been reported that in animals without histological lesions there is a chronic state of hypoperfusion in the hemisphere ipsilateral to the occlusion, where the blood flow is above the threshold of ischemia, but below normal levels [54].

In the adult's brain, the system EPO/EPO-R seems to be in a quiescent state. The role of EPO receptor in its neuroprotective action is present time topic, but it is not aim of this scientific research. Indeed, a very recent paper showed a neuroprotective effect of EPO in an EPO-R knock out stroke animal model, which suggests that EPO's neuroprotective effect is not only through the EPO-R receptor; it probably involves the regulation of CBF [55]. On the other hand, Sanchez et al. 2009 [56] reported that an optimal neuroprotectant by erythropoietin requires an elevated expression of its receptor in neurons. It was also confirmed in our previous work [57].

The expression of the system EPO/EPO-R in the brain, and specifically in areas with neurons that are very vulnerable to ischemic insult (hippocampus and cerebral cortex) has been recently demonstrated [58]. The differential stimulation of the EPO system/EPO-R rapidly after hypoxia emphasizes its role as a physiological mechanism of protection in the mammalian brain [59].

The cognitive impairment observed in the open field test can be attributed to damage in the hippocampus, cerebral cortex, and thalamus [35]. These observed structures that were neuroprotectant are supplied by arteries that are not frequently affected in ischemic injury. These arteries are on the periphery of the infarct, which appears likely in the first hours after occlusion. In this group, there was only light damage in the CA1 and CA2 regions of the hippocampus.

The intranasal route offers the possibility of administering lower doses than those required intravenously [20, 28, 60]. In addition, it allows chronic, acute, and safe treatment; which is a necessary condition that a neuroprotectant must have against the neurodegenerative illnesses.

It has been shown that treatment with Neuro-EPO immediately after occlusion in this model, is effective in reducing mortality in both sexes [32]. Previous experiments have shown that gerbils that develop clinical ischemia signs have a high incidence of pathological events in the hemisphere for 24 hours after surgery and die within 72 h [54].

One of the limitations of the neuroprotective agents assessment, in preclinical and clinical, is the therapeutic time window, in which the agent is able to interfere with the processes leading to cell death and the consequent impairment of brain function due to ischemia [58, 61]. The intravenous administration of rHu-EPO in a model of MCA occlusion in rat-neuroprotective effects extended up to 6 hours after injury [62]. This therapeutic window is twice that of tissue plasminogen activator (tPA), and the only drug treatment currently approved to treat the acute ischemic stroke and thrombosis [59, 63, 64].

It has been shown that the expression of mRNA for the formation of the molecule of EPO and its receptor takes between 2 and 4 hours after the ischemic insult. Thus, protecting endogenous small insults solves the problem, but when the flow decreases to a critical level, it is necessary to exogenous EPO administration to assist the equilibrium establishment that involves minimizing the damage caused by the ischemic cascade [65].

It has been compared in recent studies by Tsai et al. [66] and Wang et al. [67] and Villa et al. [68] that the derivative of the rHu-carbamylated EPO (CEPO) has a wide therapeutic window in focal ischemia model in rats; it also has not the effect in the stimulation of the platelets production.

In general, the results have corroborated that in Neuro-EPO has neuroprotective properties similar to asialic and carbamylated derivatives of rHu-EPO, and the intranasal administration seems to be related to a therapeutic window period as large as 12 h for the acute manifestations of ischemia in this model, or 18 hours for chronic manifestations [7, 37, 69–72]. Indeed, Neuro/EPO *IN* showed the highest therapeutic window reported in preclinical stroke studies till now.

The fundamental finding of this study has shown that the therapeutic window of the Neuro-EPO *IN* is up to 12 h, which gives this molecule very beneficial characteristics in future clinical application [69]. In medical practice, the therapeutic window is a crucial factor that determines the efficacy of neuroprotection [73].

In summary, the dose effect and the average dose used Neuro-EPO (249.4 UI/10 *μ*L/every 8 hours for 4 days) proved to be valid indicators of viability, neurological status, and spontaneous exploratory activity since it is significantly lower than the reported for the systemically use of the rHu-EPO as neuroprotectant. In addition to that, up to 12 h window for Neuro-EPO *IN* administration as a candidate for neuroprotection in acute brain ischemia is very positive quality. These solid results may support the way of this molecule to the clinical trail for the acute stroke treatment.
